# A survey of stakeholder perspectives on exoskeleton technology

**DOI:** 10.1186/1743-0003-11-169

**Published:** 2014-12-19

**Authors:** Jamie Wolff, Claire Parker, Jaimie Borisoff, W Ben Mortenson, Johanne Mattie

**Affiliations:** Occupational Science and Occupational Therapy, University of British Columbia, (T325 - 2211 Wesbrook Mall), Vancouver, V6T 2B5 Canada; Canada Research Chair in Rehabilitation Engineering Design, British Columbia Institute of Technology, (3700 Willingdon Ave.), Burnaby, B5G 3H2 Canada; MAKE+, British Columbia Institute of Technology, (3700 Willingdon Ave.), Burnaby, B5G 3H2 Canada; ICORD (International Collaboration on Repair Discoveries, 818 West 10th Avenue, Vancouver, V5Z 1M9 Canada; Principal Investigator, Rehabilitation Research Program, GF Strong Rehabilitation Centre, 4255 Laurel Street, Vancouver, V5Z 1M9 Canada; Biomedical Engineering Program, University of British Columbia, 2010-2332 Main Mall, Vancouver, V6T 1Z4 Canada

**Keywords:** Exoskeleton, Powered orthoses, Spinal cord injury, Social participation, Mobility, User perspective

## Abstract

**Background:**

Exoskeleton technology has potential benefits for wheelchair users’ health and mobility. However, there are practical barriers to their everyday use as a mobility device. To further understand potential exoskeleton use, and facilitate the development of new technologies, a study was undertaken to explore perspectives of wheelchair users and healthcare professionals on reasons for use of exoskeleton technology, and the importance of a variety of device characteristics.

**Methods:**

An online survey with quantitative and qualitative components was conducted with wheelchair users and healthcare professionals working directly with individuals with mobility impairments. Respondents rated whether they would use or recommend an exoskeleton for four potential reasons. Seventeen design features were rated and compared in terms of their importance. An exploratory factor analysis was conducted to categorize the 17 design features into meaningful groupings. Content analysis was used to identify themes for the open ended questions regarding reasons for use of an exoskeleton.

**Results:**

481 survey responses were analyzed, 354 from wheelchair users and 127 from healthcare professionals. The most highly rated reason for potential use or recommendation of an exoskeleton was health benefits. Of the design features, 4 had a median rating of very important: minimization of falls risk, comfort, putting on/taking off the device, and purchase cost. Factor analysis identified two main categories of design features: *Functional Activities* and *Technology Characteristics*. Qualitative findings indicated that health and physical benefits, use for activity and access reasons, and psychosocial benefits were important considerations in whether to use or recommend an exoskeleton.

**Conclusions:**

This study emphasizes the importance of developing future exoskeletons that are comfortable, affordable, minimize fall risk, and enable functional activities. Findings from this study can be utilized to inform the priorities for future development of this technology.

**Electronic supplementary material:**

The online version of this article (doi:10.1186/1743-0003-11-169) contains supplementary material, which is available to authorized users.

## Background

While wheelchairs may promote activities of daily living and participation in the community [1, 2], a strong desire remains for standing and walking as a means of mobility among many wheelchair users [3, 4]. Standing and walking, either independently or with assistance, may also improve several aspects of health, including blood pressure, joint range of motion, bladder health, skin integrity, spasticity, and pain [5–7]. Simple devices such as standing frames offer several of these benefits [6]. Clinical gait training with body weight-support, either therapist assisted or using robotic devices such as the Lokomat, is becoming more widespread due to its health and rehabilitation benefits [8, 9]. Technological efforts to enable functional ambulation (i.e. to replace the wheelchair) have been underway for decades. Orthotics such as long leg braces are still prescribed, although they are rarely used by people with spinal cord injury (SCI). Newer passive orthoses, such as the reciprocating gait orthosis [10, 11] and hip guidance orthosis [12] have been developed; however, their use is also limited. The latest efforts concern the development of robotic exoskeletons.

A robotic exoskeleton is a wearable, powered lower limb orthosis that uses a system of actuators and sensors to achieve walking movements. Currently exoskeletons are primarily used in supervised clinical settings for health and rehabilitation purposes, but are eventually intended for daily use as a functional mobility device [13]. The ReWalk™ exoskeleton was recently approved for home use by the United States Food and Drug Administration, when the user is accompanied by a specially trained assistant [14]. Most current designs (such as the ReWalk™, Ekso Bionics™, and Indego™) require the use of arm crutches or a walker for stability. The Rex™ robotic walking device, however, is self-supporting, requiring no other device for stability. Exoskeleton users initiate movement either with hand controls or using the position of their upper body. Primary candidates for this type of technology are individuals with mobility impairments, in particular those who rely on wheelchairs for mobility and have bilateral upper extremity function. Approximately 0.6% of Canadians (210,000 people) and 0.7% of Americans (2.2 million people) reported using a wheelchair, in 2006 and 2012 respectively [15, 16]. Many of these individuals could, therefore, be potential candidates to use an exoskeleton.

Exoskeletons may play a larger role in rehabilitation moving forward [9]. A recent narrative review found that using exoskeletons as a method of partial assistance for rehabilitation following incomplete spinal cord injury was an effective technique for gait retraining and strengthening functioning muscles [17, 18]. Further, a systematic review on exoskeletons in stroke rehabilitation found that their use in combination with physiotherapy led to an increased incidence of independent walking [19]. Two studies examining safety training and tolerance for the ReWalk™ exoskeleton over short distances demonstrated it had low safety risks, was well tolerated, and that users improved in its use with training [20, 21]. Spungen et al. [13] noted that with training, some participants were able to independently perform selected home and community based skills using the exoskeleton, including walking on a slope and accessing a high shelf while standing.

While there is much excitement around these new robotic exoskeletons, there are issues that may limit their utility both as a therapeutic device and as a mobility device. Some significant limiting factors include difficulty donning and doffing, problems transferring, slow and often rough movement, lack of dependability, and concerns surrounding pressure distribution and skin integrity [22]. Researchers have identified four key topics for future development of exoskeletons: robust control, safety and dependability, ease of wear-ability or portability, and usability/acceptance [23]. For example, if a person cannot easily use a device, or has problems with accepting a novel technology, it will likely be abandoned or not used to its full potential [22]. For this reason, the wider acceptance of exoskeletons for both rehabilitation and function is dependent on the end user being central to design and development of the technology [23].

Despite the potential benefit of these devices, and importance of user acceptance, little is documented about stakeholder perspectives on exoskeletons. One qualitative study found that potential end users and mobility specialists were primarily concerned with the safety, cost, ease of use, and functionality of the device [24]. Additional research on user perspectives and applicability of exoskeletons is needed in order to understand the features that stakeholders feel are most important, in order to guide development of safe, functional, user-friendly devices. Therefore this study was undertaken to examine and compare stakeholder (wheelchair users and healthcare professionals) perspectives on exoskeleton technology, with respect to perceived importance of design features and potential reasons for use.

## Methods

### Study design

Data for the study were collected using an online survey, which was developed and administered using the tailored design method [25]. The survey was piloted to a small group of participants (n = 6), from both stakeholder groups. Based on their feedback, minor adaptations were made to wording and layout, and a final version of the survey was created, which was approved by the Behavioural Research Ethics Board of the University of British Columbia.

The survey included 30 questions: 7 questions to collect demographic information (age, sex, country of residence, level of education, primary diagnosis, and profession); 5 questions related to past experiences and familiarity with exoskeleton technology; and 17 questions about reasons for use of an exoskeleton and importance of various design and functionality considerations. Questions primarily used a multiple choice response format (demographics and reasons for use), or a 5-point Likert scale, with Likert scales ranged from 1-Very Unimportant to 5-Very Important (design considerations), or 1-Strongly Disagree to 5-Strongly Agree (statements about exoskeleton design characteristics). Participants were asked to respond Yes or No to whether they would use an exoskeleton for: health benefits, rehabilitation purposes, social interactions, and/or functional day-to-day tasks. Participants were also asked one open-ended question. Wheelchair users were asked: “Are there any other reasons you would use an exoskeleton?”, whereas healthcare professionals “Are there any other reasons you would recommend an exoskeleton?” The full survey is available as Additional file 1.

### Sample

Two groups of stakeholders were recruited for this study: wheelchair users and healthcare professionals working directly with wheelchair users. To be eligible for this study, wheelchair users needed to be over 18 years of age, fluent in English, and use a wheelchair as a primary means of mobility (self-defined). Healthcare professionals needed to be over 18 years of age, fluent in English, and have experience working with individuals with mobility impairments (e.g., occupational therapists, rehabilitation assistants, nurses, physiotherapists, physiatrists, orthotists, assistive technology specialists, or mobility equipment vendors). Because the study aimed to evaluate perspectives on potential rather than actual use of the devices in question, no exclusion criteria related to participants’ physical abilities were set.

Participants were recruited using mass emails to databases of research volunteers from prior studies conducted by the authors’ respective organizations, postings on user and healthcare professional online forums, flyers posted in rehabilitation centres, social media, and word of mouth. Data were collected between February and June, 2014.

### Data analysis

The quantitative data were analyzed using IBM SPSS Statistics for Windows, Version 22.0. Armonk, NY: IBM Corp. Descriptive statistics and graphic representations were used to characterize the sample and to compare the importance of different design features. Importance comparisons were conducted using medians, and % of respondents rating a factor as “important” or “very important”.

Exploratory factor analysis (EFA) was used to examine how responses to individual questions about different exoskeleton design characteristics (i.e. variables) were related to one another. That is, could the characteristics be grouped together into certain categories? EFA is a method to extract these broad underlying categories, which are then called factors [26]. The number of factors to be extracted was determined through examination of a scree plot of the eigenvalues [26]. Maximum likelihood was the method of extraction and direct oblimin with Kaiser normalization was the method of rotation. For factor analysis, loadings > .71 are considered excellent, >.63 are considered very good, >.55 are considered good, and > .45 are considered fair [27].The Kaiser-Meyer-Olkin (KMO) measure and Bartlett’s test of sphericity were utilized to ensure adequacy of the sample for EFA [26]. For the KMO measure, a minimum of 0.5 is recommended, 0.60-0.69 is considered mediocre, 0.70-0.79 is considered fair and 0.80-0.89 is considered good [28].

Through this method, associations and patterns among groups of variables were used to group potential exoskeleton features into factor-based categories. These categories were then compared for wheelchair users and healthcare professionals using an independent samples Mann–Whitney U test. Chi-squared tests were performed to determine significant differences between stakeholder groups for reasons to use or recommendation of an exoskeleton.

Responses to open-ended questions were analyzed using content analysis [29]. Analysis was based in a perspective of engagement in meaningful activity, and themes organized using the Human Activity Assistive Technology (HAAT) model [30]. This model describes a person, an activity, and assistive technology interacting within a context [30]. The HAAT model depicts the person as possessing underlying skills and abilities which they bring to a given task. The assistive technology, in this case the exoskeleton, influences human performance. This occurs within a context, which includes the physical, social, and cultural environments. This framework was used as an analytical lens to conceptualize the multifaceted nature of the human-technology interaction within themes. Emergent coding (i.e.,exploring the content without previously formulated assumptions about the results) was used to establish categories from the individual responses, and inductive analysis, generating broader ideas based in specific details, was performed to combine categories into broader themes [29]. Relative frequencies of categories and themes were assessed to determine the most prevalent themes within the responses. Responses could be coded with more than one theme.

## Results

### Participants

A total of 603 participants responded to the survey. Of these, 122 respondents did not meet the inclusion criteria and/or did not fully complete the survey and were excluded from the data analysis. Demographic information about the 481 remaining respondents is described in Table 1.

**Table 1:** **Demographics of stakeholder groups**

Wheelchair Users (n = 354)	Frequency	Percent
*Gender*		
- Male	194	54.8%
- Female	160	45.2%
*Age*		
18-24	17	4.8%
25-34	59	16.7%
35-44	70	19.8%
45-54	93	26.3%
55-64	72	20.3%
65 and above	43	12.1%
*Country*		
- Canada	197	55.6%
- United States	129	36.4%
- Other	27	7.6%
*Diagnosis*		
- SCI (paraplegia)	130	36.7%
- SCI (quadriplegia)	87	24.6%
- MS	30	8.5%
- CP	24	6.8%
- Muscular Dystrophy	19	5.4%
- Post-polio	13	3.7%
- Congenital SCI	12	3.4%
- Stroke	10	2.8%
- Other	32	9.0%
*Hours per day using a wheelchair*		
0–4 hours	35	9.9%
5–8 hours	40	11.3%
9–12 hours	86	24.3%
12+ hours	193	54.5%
*Previous use of an exoskeleton*		
No	328	95.6%
Yes	15	4.4%
**Healthcare Professionals (n = 127)**	Frequency	Percent
*Gender*		
- Male	44	34.6%
- Female	83	65.4%
*Country*		
- Canada	76	59.8%
- United States	41	32.3%
- Other	10	7.9%
*Profession*		
Occupational Therapist	25	19.7%
Physiotherapist	21	16.5%
Equipment vendor	13	10.3%
Nurse	9	7.1%
Support staff	8	6.3%
Rehabilitation assistant	7	5.5%
Rehabilitation engineer	7	5.5%
Clinic director/manager	6	4.7%
Assistive technology specialist*	5	3.9%
Research professional	5	3.9%
Physician	3	2.4%
Orthotist	2	1.6%
Other**	16	12.6%
*Previous use of an exoskeleton*		
No	108	93.1%
Yes	8	6.9%

Breakdown of characteristics of the 481 respondents, by stakeholder group.

*This category included job titles such as seating specialist, AT provider

**This category consists of specific job titles of which there were two or fewer incidences which could not be grouped into the other categories, e.g. social worker, disability services provider.

### Reasons to use an exoskeleton

**Figure 1:**
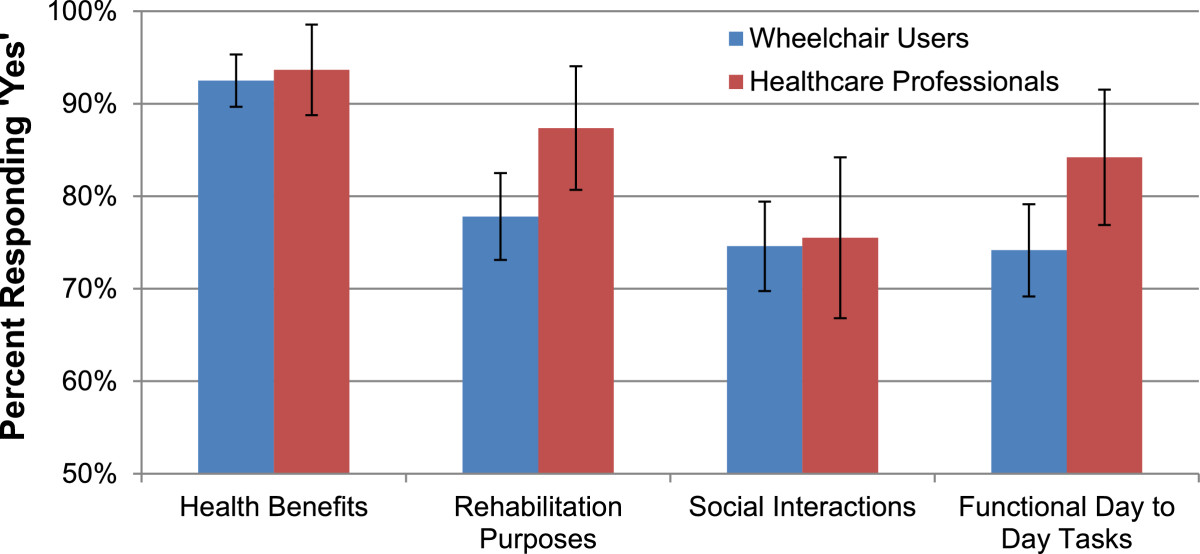
**Reasons to use of recommend an exoskeleton.** Participants were asked to respond “Yes” or “No” to whether they would use or recommend an exoskeleton for health benefits, rehabilitation purposes, social interactions, and functional tasks. Health benefits was the most commonly supported reason by both stakeholder groups. Error bars denote 95% confidence intervals.

When participants were asked whether they would use an exoskeleton for health benefits, rehabilitation purposes, social interactions, and/or functional day-to-day tasks, the reason most frequently rated “yes” was health benefits (See Figure 1). Specific potential health benefits identified by respondents included pressure relief, increased circulation, improved bone density, improved bowel and bladder function, reduced risk of orthostatic hypotension and general benefits associated with standing and walking.

Stakeholders were asked to agree or disagree (1-Strongly disagree to 5-Strongly agree) on three additional statements about exoskeleton use. Wheelchair users agreed with two statements significantly more than healthcare professionals: “A powered exoskeleton is a good idea” (Chi-Square = 14.885, p = 0.005) and “I would use/recommend an exoskeleton” (Chi-Square = 31.316 p = 0.001), although the majority from both groups had agreement with both statements. Conversely, more healthcare professionals thought users would feel self-conscious using the device in public compared to wheelchair users (Chi-Square = 35.067 p = 0.001).

### Design features

Participants ranked 17 design features on a Likert scale from 1 (Very Unimportant) to 5 (Very Important). Descriptive statistics were used to illustrate the differences in importance between the features (see Table 2). When considering all participants as one group, four of the 17 potential design features were rated with a Median of 5 (i.e. “very important”): minimizes risk of falling, purchase cost, comfort, and putting on/taking off the device. Appearance and length of training time were overall rated lowest with a Median of 3 “neither important nor unimportant”.

**Table 2:** **Importance of exoskeleton design features**

Exoskeleton design features	Mean importance	Standard deviation	Median importance
Minimizes risk of falling	4.54	0.828	5
Purchase cost	4.39	0.912	5
Comfort	4.38	0.838	5
Repair and maintenance cost	4.34	0.844	4
Ease of putting on and taking off the device	4.25	1.033	5
Range of battery life	4.23	0.859	4
Ability to walk on uneven surfaces	4.22	0.922	4
Amount of energy needed for use	4.15	1.015	4
Ability to carry out daily tasks while standing	4.13	0.946	4
Portability of the device	4.09	0.942	4
Ability to toilet	4.05	1.071	4
Ability to use to get in and out of a car	3.97	1.033	4
Ability to climb stairs	3.91	1.029	4
Ability to use without arm crutches	3.71	1.006	4
Walking speed	3.64	0.985	4
Length of training to become proficient	3.34	1.082	3
Overall appearance	3.23	1.177	3
**Valid N = 405**			

Descriptive statistics used to illustrate the difference in importance between ratings of 17 potential design features. These features were ranked by respondents on a Likert scale from 1 – Very Unimportant, to 5 – Very Important.

**Figure 2:**
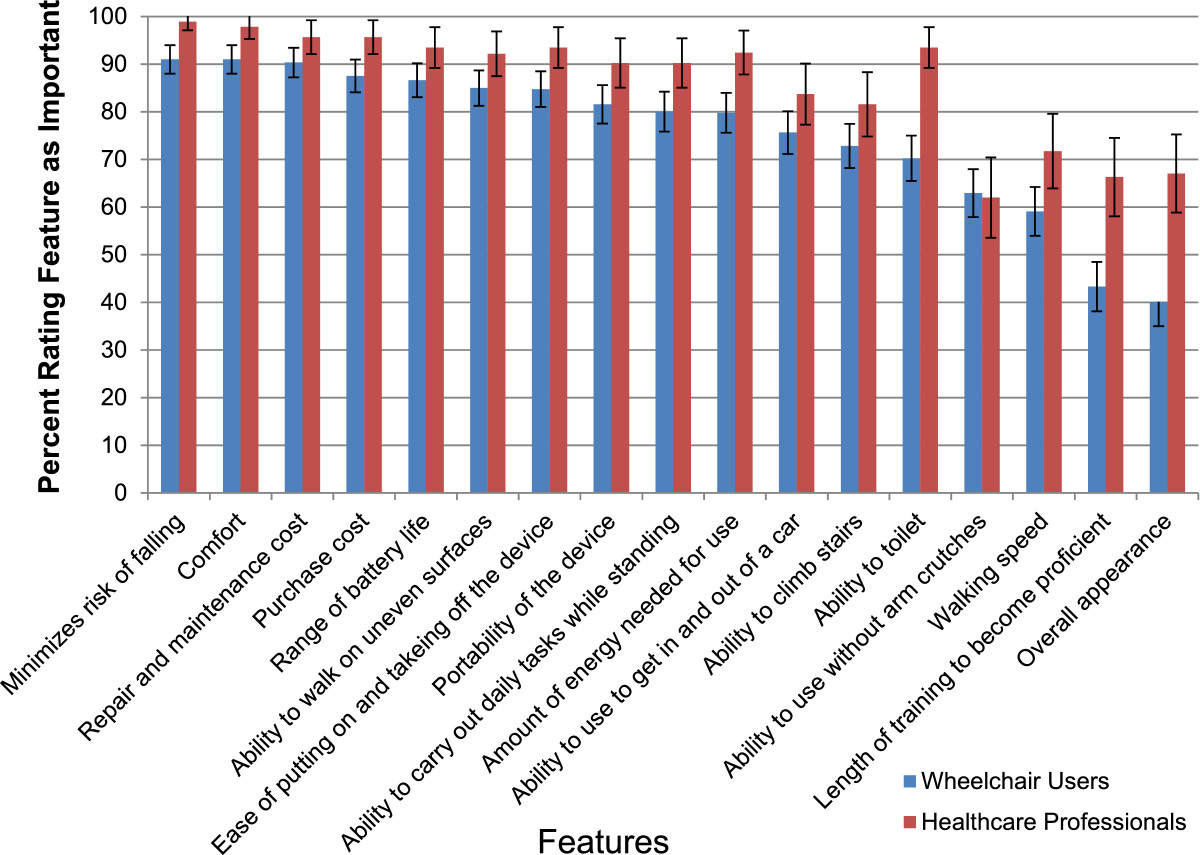
**Importance of design features.** 17 design features were ranked on a Likert scale from 1 – Very Unimportant, to 5 – Very Important. The percentage of respondents who identified features as either ‘4 - Important’ or ‘Very Important’ is shown. Healthcare professionals tended to rate all features as more important than their wheelchair user counterparts. Error bars denote 95% confidence intervals.

To help compare which design features were most important across both groups, the percentage of participants rating each feature as important or very important on the Likert scale was calculated. Comfort, minimizes risk of falling, repair and maintenance cost, and purchase cost was rated as important or very important by between 75 and 80% of all participants. Six other features were rated important by between 70 and 74% of participants: range of battery life, ease of putting on and taking off, ability to walk on uneven surfaces, portability of the device, amount of energy needed for use and ability to carry out daily tasks while standing.When the stakeholder groups were examined separately, a similar trend to the overall data was evident in each group. However, some features showed a discrepancy in opinion between stakeholder groups. One discrepancy was when asked to identify an appropriate price range for a powered exoskeleton, the median reported price by healthcare professionals was $10,000-$20,000USD, compared to the median reported by wheelchair users of under $10,000USD. An overall trend when comparing design features was that healthcare professionals rated every feature as more important than did wheelchair users, with the exception of the ability to walk without arm crutches. Additionally, variance was larger for wheelchair users than health care professionals for all importance questions. Figure 2 shows the relative importance of all 17 features to both stakeholder groups.

### Exploratory factor analysis

Exploratory factor analysis found two underlying factors that encapsulated the 17 question items regarding the importance of different potential features of the technology. Of the 17 items, 8 items loaded onto factor 1, and 9 items loaded onto factor 2. All but two items loaded as fair or above, defined as > .45 (see Table 3). Cross-loading, defined as a variable which loads as > .30 on both factors [26], was evident for two items: portability and battery life/range. However, both items loaded more strongly onto factor 2. Overall appearance did not load well onto either factor, although it loaded more strongly onto factor 1.

**Table 3:** **Exploratory factor analysis**

Exoskeleton design features	Factor 1 (Technology characteristics)	Factor 2 (Functional activities)
Purchase cost	*0.778*	
Repair and maintenance cost	*0.758*	
Comfort	*0.701*	-0.128
Ease of putting on and taking off the device	*0.694*	
Minimizes risk of falling	*0.659*	-0.107
Amount of energy needed for use	*0.659*	
Length of training to become proficient	*0.509*	
Overall appearance	*0.375*	-0.107
Ability to climb stairs	-0.212	*-0.855*
Ability to carry out daily tasks while standing		*-0.757*
Ability to use to get in and out of a car		*-0.731*
Ability to walk on uneven surfaces	0.110	*-0.672*
Walking speed		*-0.573*
Ability to toilet	0.142	*-0.495*
Portability of the device	0.395	*-0.471*
Range of battery life	0.310	*-0.459*
Ability to use without arm crutches	0.171	*-0.419*

Associations (i.e. loadings) of individual design features and the two factors (i.e. categories) are revealed through exploratory factor analysis. Higher numbers indicate a stronger association between the design feature (variable) and the factor, where > .71 are considered excellent, >.63 are considered very good, >.55 are considered good, and > .45 are considered fair. Values between -0.100 and 0.100 have been excluded from this table. These loadings allow the design features to be grouped into two major categories, where Factor 1 represents Technology Characteristics and Factor 2 represents Functional Activities. Italicized loading values indicate the factor which the design feature was grouped into.

Factor 1 included items generally related to device characteristics (Factor 1 was labeled *Technology Characteristics*), whereas Factor 2 included items related to activities and tasks (Factor 2 was labeled *Functional Activities*). Overall, *Technology Characteristics* were rated as slightly more important than *Functional Activities* (Mean = 4.078, SD = 0.689 and Mean = 3.995, SD = 0.706 respectively). Sampling adequacy was good as determined by the KMO measure (KMO = 0.903) [26]. Bartlett’s test of sphericity indicated that correlations between items were sufficiently large for factor analysis, Chi-Square = 3577.059, p < 0.001.

Independent samples Mann–Whitney U tests were used to determine differences in perceived importance of each factor across stakeholder groups. Importance of *Technology Characteristics* (Factor 1) and importance of *Functional Activities* (Factor 2) both varied significantly between wheelchair users and healthcare professionals (U = -4.651, p = 0.001 and U = -2.288, p = 0.022, respectively). In both cases the healthcare professionals rated these factor as more important than wheelchair users.

### Qualitative analysis

Content analysis of the open-ended questions regarding further reasons to use or recommend an exoskeleton showed consistent underlying themes both within and between groups (See Table 4). Response rate to the open-ended questions was 47.7% of total wheelchair user survey respondents and 33.9% of total healthcare professional survey respondents.

**Table 4:** **Qualitative themes**

Theme*	Associated categories
*Psychosocial Benefits*	Roles & relationships, psychological, quality of life, independence, eye-level social interaction, curiosity/interest, “cool”, social, experience
*Health and Physical Benefits*	Health, pressure management, pain control, walking, standing, exercise, transfers, rehabilitation
*Uses in Daily Life*	Leisure, employment, functional day-to-day tasks, access, outdoor use
*Larger Impacts*	Research & development, visibility, advocacy
*Client-driven*	Client goals, motivation, use of available resources
*Device will not work*	Potentially harmful, inefficient, impractical, too expensive, dislike aesthetic
*Not compatible with my impairment*	Hemiplegia, quadriplegia, low bone density, contractures, lack of arm/hand use, poor balance, amputee, obesity, muscular dystrophy, uneven lower extremities

Themes derived from responses to the open-ended question “Are there any other reasons you would use/recommend an exoskeleton?” using content analysis. A full copy of responses is included as an additional file.

*Themes are ordered by prevalence within the qualitative responses.

**Figure 3:**
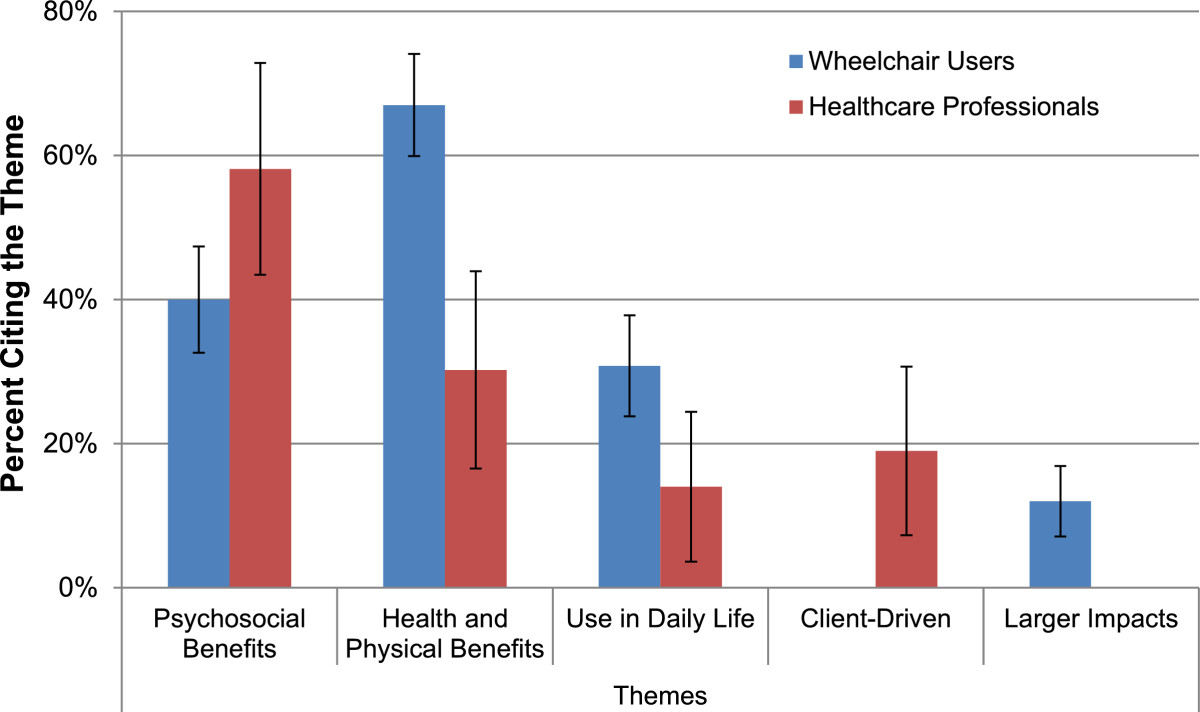
**Qualitative themes.** Themes derived from open-ended question responses using content analysis. Total n for this question was 169 WC users and 43 HCPs. Respondents could cite more than one theme within an answer. Error bars denote 95% confidence intervals.

The themes identified among both stakeholder groups are illustrated in Figure 3. Four major categories from the HAAT model were represented in the themes: Person, Activity, Context, and Assistive Technology [30]. Three common themes were found in both wheelchair user and healthcare professional populations. Psychosocial Benefits (Person) was the most common theme identified by healthcare professionals and the second most common theme identified by wheelchair users. One participant responded, “I’d like to stand and kiss my husband, I’d like to meet people eye to eye again, I’d like to breathe the air up there”. The Health and Physical Benefits (Person) theme was the most prevalent theme represented in the responses of wheelchair users and second for healthcare professionals. One wheelchair user noted, “The health benefits alone would be worth it”. A healthcare professional replied that they perceived the device’s benefits to be “mostly for health and rehab”. The third theme found in both groups was Use in Daily Life (Activity, Context) and included functional and accessibility considerations. One wheelchair user respondent noted, “More independence in getting around a community not structured for wheelchair users”, and another, “Try doing the dishes, cook delicious meals for my family… walking up and down the stairs in my own beautiful home”. One theme unique to wheelchair users was Larger Impacts (Context), i.e., using exoskeleton technology as a means of contributing to development, or as a method of advocacy and visibility for individuals with disabilities. A theme unique to healthcare professionals was “Client-driven” (Person) and included exoskeleton use because of client interest, or as a method of motivating clients in the rehabilitation process. One healthcare professional respondent noted, “Motivation during the rehab process. It would be more exciting for a patient to use an exoskeleton during therapy to walk somewhere instead of on a treadmill, like the Lokomat or other similar devices”.

Two final themes related to potential problems using the device. Some respondents felt that the device would not work (Technology) for reasons such as impracticality, inefficiency, a potential for harm, and an inherently high cost that would prohibit many individuals from use. Respondents posed questions such as “I’m really curious if you fall, what can and will go wrong?” and “Have you considered the pressure sore issues that could result?”. A second group of respondents felt while the device may have merit, they personally would not use it (Person, Technology). In both groups, this was predominantly due to the inability to use the device given their (or their clients’) impairment (examples given included: high quadriplegia, hemiplegia, joint contractures, and low bone density), although some users were simply not interested in walking in such a device. For a complete report of all participant responses to the open-ended questions, please see Additional file 2.

## Discussion

### Perspectives on exoskeletons

This is one of the first studies to examine the perspectives of healthcare professionals and potential end-users on exoskeleton technology. Previous research on adoption of assistive technology devices in general has also identified safety and cost as priorities for users [31]. Our study expands these findings to conclude that these same features are important to both users and healthcare professionals when considering exoskeletons specifically. These features are in line with interactions between the person and their assistive technology within their context of use, as described by the HAAT model [30]. These considerations are important to the use and adoption of the technology [32].

Two of the most considered factors in recent research regarding exoskeletons are comfort and safety. Contemporary studies have focused on falls risk as well as other safety concerns of the device, such as proper fit in order to maximize comfort and minimize pressure areas [20, 21, 33, 34]. Safety was also identified by users in a study by Matthews et al. [31] as a primary concern for any assistive technology. Within our study, concerns were raised by respondents within the open-ended questions that the technology had potential for harm, both in terms of pressure issues and falls risk, and that wheelchairs remained a safer, more effective option. While current trials of exoskeletons show low safety risks [20, 21], these are in supervised clinical settings with a trained therapist guarding the user from falls. If exoskeletons are to be used for functional activities, this will be in less controlled environments and may require some trade-off between the safety and overall function of the devices. Additionally, device developers may look to proactively design technology to mitigate falls in unsupervised settings.

Cost was identified by users as a potential concern in previous research examining reasons for choosing to adopt assistive technology [31]. Currently, purchasing a ReWalk exoskeleton for personal use costs just under $70,000USD [35], substantially higher than the reported acceptable cost in this study of under $20,000USD. Some survey respondents reported that they would not use an exoskeleton due to the fact that it may cost too much to purchase and maintain as a personal device. Similar contextual and economic barriers were identified by a recent study assessing the adoption of robotics in rehabilitation; cost was one of the largest concerns raised in this study, due in part to the unknown cost-effectiveness of robotic devices [32]. Our findings also show that stakeholders have similar concerns with both the purchase and maintenance costs of exoskeletons.

Features of the technology were grouped by exploratory factor analysis into two separate categories of design features which resonated with two components of the HAAT model, Activity and Assistive Technology [30]. These factors were named *Functional Activities* and *Technology Characteristics*. In the HAAT model, the two components interact with the person and their context, providing a comprehensive understanding of how multifaceted the user-assistive technology relationship can be. Our study results show a similar relationship was perceived by potential stakeholders of exoskeletons.

There were some small but significant differences between the importance of the two categories when compared by stakeholder group. *Technology Characteristics* were slightly more important to health care professionals, which may be related to the current use of exoskeletons mainly for health benefits and rehabilitation where the clinical setting necessitates significant involvement from the healthcare professional. Therefore, technology characteristics which support rehabilitation would be necessary when attempting to integrate exoskeletons into their practice [32]. In the current clinical context, it may be more important to consider the perspectives of healthcare professionals, as they mediate most present use of exoskeletons. In future, wheelchair users’ perspectives may become more salient as the devices move towards individual, functional use. It is also worthwhile to acknowledge that both factors fell within the range of “important” to both stakeholder groups. This would indicate that a multifaceted perspective on development of exoskeletons is key; stakeholders are invested both in the design of the technology, as well as what the technology enables users to accomplish.

Health benefits of standing and walking are frequently identified in current literature, a perception which appears to be mirrored in the perceptions of stakeholders in this study [5, 6]. This may be reflective of health benefits being the most studied benefit of exoskeletons in their current form. It could also reflect the priorities of users and healthcare professionals in seeking to optimize physical health for better long-term outcomes.

Psychosocial benefits, though not well documented in the literature, were also noted as a perceived benefit to the use of exoskeletons. Opportunities for eye-level social interaction, and the joy, hope, and confidence that users felt that standing and walking could bring them were identified by several wheelchair users. This shows that the potential benefits of standing and walking, especially outside of the clinical rehabilitation setting, can include psychosocial as well as physical benefits. Healthcare professionals rated recommending an exoskeleton for psychosocial reasons more highly than wheelchair users, which could potentially relate to the health care professional Client-Driven theme identified here, specifically, targeting motivation and psychosocial benefits to accomplish physical goals.

The potential for use as a rehabilitative device was identified as a further reason for use of an exoskeleton. Use of exoskeletons in rehabilitation settings for SCI and stroke populations has been portrayed positively in the literature, however, this reason was not as frequently stated in this study’s quantitative and qualitative results when compared to health benefits and social interactions [17, 19]. This may be a reflection of the narrow potential user population that would meet the physical requirements to both use an exoskeleton and have the potential to benefit from its rehabilitative effects (e.g., incomplete paraplegia).

Though use of an exoskeleton for functional daily tasks was identified as a potential reason for use, it was rated lower than others. This may be due to the current limitations of the technology, which includes a relatively slow walking speed. However, some wheelchair users appeared to have higher expectations than are feasible with current technology. This perception creates a potential discord with the realistic functional benefits of using the device. Examples of this included a number of responses from users who felt that an exoskeleton would enhance independence in daily life. Many stakeholders also identified ease of putting on and taking off the device as an important consideration. While these functions may be limited with the current technology, they can, nevertheless, provide direction towards the design of desirable features or functions of future exoskeletons.

### Implications for future developments

If exoskeletons are to be adopted as mainstream mobility devices, additional research and development is required to enhance the affordability, comfort, safety, and ease of use of exoskeletons to achieve stakeholder goals. Other areas of attention are also surely important to stakeholders. However, to reduce participant burden in this study, some, more detailed questions, were not included. Further study into areas such as specific falls prevention and/or recovery strategies, specifics of hardware and control designs, and directly addressing how the device could control for issues related to spasticity, contractures, or other individual needs is indicated going forward. Many wheelchair users expressed interest in using the devices to increase visibility and advocacy. This shows the readiness and willingness of the wheelchair user community to engage in and support development of new technology, which is invaluable for developers and researchers. As exoskeleton development continues, it will be important to re-evaluate and expand on stakeholder perspectives to maximize their utility and adoption [32].

### Study limitations

The study had four main limitations. Firstly, the format of an online survey limited the sample to those individuals who had access to a computer and who were fluent in English. Secondly, participants were primarily from North America. These may have resulted in issues with how representative the sample is of the broader population. A volunteer bias may have impacted the types of responses. The voluntary nature of participation in an online survey means that it is likely that participants already had some interest or opinion on exoskeleton technology. It is also possible that there was a social desirability bias to respond positively towards questions about exoskeletons [36].

## Conclusions

An online survey was conducted to determine stakeholder perspectives on exoskeleton technology. Wheelchair users and health-care professionals reported that there could be potential health, psychosocial, and functional benefits to the use of exoskeletons. They also identified safety, purchase cost, maintenance costs, ease of use, and comfort as very important when considering whether or not they would use or recommend this type of device. Several other features were also identified as important. Features relating to functional activities and characteristics of the technology were both identified as important by healthcare professionals and wheelchair users, indicating the need to address both in exoskeleton research and development. Findings from this study lay groundwork for future research into stakeholder perspectives on exoskeleton technologies, aiming to inform the ongoing development of these devices in a user-centred direction.

## Electronic supplementary material

Additional file 1: Survey questions. (DOCX 306 KB)

Additional file 2: Responses to open-ended questions. (DOCX 46 KB)

## Abbreviations

BCIT: British Columbia Institute of Technology
CP: Cerebral palsy
EFA: Exploratory factor analysis
HAAT: Human Activity Assistive Technology
ICORD: International Collaboration on Repair Discoveries
KMO: Kaiser-Meyer-Olkin measure
MD: Muscular dystrophy
MS: Multiple sclerosis
RA: Rehabilitation Assistant
RESNA: Rehabilitation Engineering and Assistive Technology Society of North America
SCI: Spinal cord injury
UBC: University of British Columbia.
