# Supplementary material for: A survey of stakeholder perspectives on exoskeleton technology
Source: J Neuroeng Rehabil. 2014 Dec 19;11:169. doi: 10.1186/1743-0003-11-169 (PMC4320449; doi:10.1186/1743-0003-11-169)
Supplement: Supplementary file 1 — Additional file 1: Survey questions. (DOCX 306 KB) [file 12984_2014_700_MOESM1_ESM.docx]

Exoskeleton

# Demographics

# 1. Do you currently work clinically with people with mobility impairments?

(i.e. mobility equipment vendor, occupational therapist, orthotist, physician, or physiotherapist)

|  | Yes (Go to Health Care Provider section: Question 3 page 11) |
| --- | --- |
|  | No |

# 2. Are you currently a wheelchair user?

|  | Yes (Continue to Wheelchair user section: Question 3 page 1) |
| --- | --- |
|  | No (Survey Ends) |

***If respondent answers that they are a wheelchair user fluid surveys directs them to the following questions designed for wheelchair users. If the respondent answers that they are not a wheelchair user and are a health care professional the survey automatically redirects them to page 12 beginning the questions designed for health care professionals.***

# Questions for wheelchair users:

# 3. What is your age?

(In years)

|  | Under 18 |
| --- | --- |
|  | 18-24 |
|  | 25-34 |
|  | 35-44 |
|  | 45-54 |
|  | 55-64 |
|  | 65 or Above |
|  | Prefer Not to Answer |

# 4. What is your gender?

|  | Male |
| --- | --- |
|  | Female |
|  | Transgender |
|  | Other |
|  | Prefer not to disclose |

# 5. What country do you currently live in?

(Drop-down menu of countries)

# 6. Please select the highest level of education you have completed.

|  | Primary or elementary school |
| --- | --- |
|  | High school |
|  | College or trade school |
|  | University undergraduate |
|  | Post-graduate |
|  | Doctor of Medicine (or equivalent) |
|  | Other (please specify) ______________________ |

# 7. What is your primary reason for using a wheelchair?

|  | Stroke (e.g. cerebral vascular accident) |
| --- | --- |
|  | Traumatic brain injury |
|  | Multiple sclerosis |
|  | Muscular dystrophy |
|  | Cerebral palsy |
|  | Arthritis |
|  | Post-polio |
|  | Spinal cord injury (Please specify the level if known) ______________________ |
|  | Congenital spinal cord abnormality (e.g. spina bifida) (Please specify the level if known) ______________________ |
|  | Other (please specify) ______________________ |

# 8. How many hours per day do you use your wheelchair?

(Hours per day)

|  | 0-4 |
| --- | --- |
|  | 5-8 |
|  | 9-12 |
|  | 12+ |

# 9. Do you use your wheelchair...

|  | Yes | No |
| --- | --- | --- |
| At your place of residence? |  |  |
| At school or in your workplace? |  |  |
| For social or recreational purposes? |  |  |
| For general mobility in the community? |  |  |

# Exoskeleton questions

# 10. How familiar are you with powered exoskeleton technology?

|  | I have never heard of it (completely unfamiliar) |
| --- | --- |
|  | I am somewhat familiar with the concept |
|  | I am very familiar with the concept |
|  | I would consider myself an expert (completely familiar) |

# 11. Have you ever used a powered exoskeleton?

|  | Yes |
| --- | --- |
|  | No |

# 12. Have you used any other standing device? (e.g. standing frame, standing wheelchair)

|  | Yes |
| --- | --- |
|  | No |

# Exoskeleton Questions

The next set of questions has to do with exoskeleton technology. Please read the description and watch the video below, then answer the following questions regarding your perspectives on the device as shown.

A powered walking exoskeleton is a mobility machine consisting primarily of an outer framework worn by a person, and a powered system of motors that delivers energy for limb movement. The purpose is to improve or replace standing or walking movements for people with mobility impairments. Most current designs require the use of arm crutches for stability, and the user can initiate movement either with hand controls, or using the position of their upper body.

Research has shown health benefits associated with standing and walking for people with a mobility impairment, such as improved: bone density, bowel and bladder function, respiration, and circulation. As well, standing and walking can reduce spasticity and tone, help to prevent pressure sores and contractures, and enhance independence and psychological wellbeing.

*Note: The video is silent.

Exoskeleton Video from Claire Parker on Vimeo: http://vimeo.com/81864531

# 13. Is walking with an exoskeleton something that interests you?

|  | Yes |
| --- | --- |
|  | No |
|  | I would need more information |

# 14. Considering the exoskeletons shown above, would you be interested in using an exoskeleton for...

|  | Yes | No |
| --- | --- | --- |
| Health benefits (e.g. improved circulation, improved bone density, decreased spasticity)? |  |  |
| Rehabilitation purposes (e.g. becoming better at walking)? |  |  |
| Functional day to day tasks? |  |  |
| Social interactions? |  |  |

# 15. Are there any other reasons you would be interested in using an exoskeleton?

# Exoskeleton questions

Current exoskeleton designs (such as those shown in the video) have a number of limitations. For example, slow movement speed, battery capacity (i.e. travel range), portability, and ease of donning/ doffing pose challenges for its use as a mainstream mobility device. As technology continues to improve, it may be possible to improve on many of these considerations.For the next set of questions, please consider both current and potential future designs; for example, which factors would be most important to improve upon.

# On the following scale from very unimportant to very important, please rate the importance of each of the following factors in consideration of whether you would use an exoskeleton, from your perspective as a wheelchair user.

# 16. Usability and Aesthetic Considerations

|  | Very unimportant | Unimportant | Neither important nor unimportant | Important | Very Important |
| --- | --- | --- | --- | --- | --- |
| Ease of putting on/taking off |  |  |  |  |  |
| Amount of energy required to use the device |  |  |  |  |  |
| Length of training (time) needed to become proficient |  |  |  |  |  |
| Overall appearance of the device itself |  |  |  |  |  |

# 17. Functional Considerations

|  | Very unimportant | Unimportant | Neither important nor unimportant | Important | Very important |
| --- | --- | --- | --- | --- | --- |
| Walking speed (getting from A to B quickly) |  |  |  |  |  |
| Range (battery life) |  |  |  |  |  |
| Ability to climb stairs |  |  |  |  |  |
| Ability to toilet while wearing the device |  |  |  |  |  |
| Ability to use without arm crutches for balance |  |  |  |  |  |
| Ability to carry out daily tasks while standing |  |  |  |  |  |
| Portability (e.g. weight, ability for the device to be dismantled for transport) |  |  |  |  |  |
| Ability to use getting in and out of a car |  |  |  |  |  |
| Ability to walk on sloped or uneven surfaces |  |  |  |  |  |

# 18. Practical Considerations

|  | Very unimportant | Unimportant | Neither important nor unimportant | Important | Very important |
| --- | --- | --- | --- | --- | --- |
| Purchase cost |  |  |  |  |  |
| Repair and maintenance costs |  |  |  |  |  |
| Minimizes risk of falling while walking |  |  |  |  |  |
| Comfort (e.g. heat, moisture, pain, stability) |  |  |  |  |  |

# 19. On the previous page, you ranked the following factors as "important" or "very important" when considering whether or not you would use an exoskeleton. Please select your top 3 most important factors from the following list.

Rank these 3 factors as 1 (the most important) to 3 (the least important) and leave the rest blank.

| Ease of putting on/taking off | \| 1 2 3 \| \| --- \| |
| --- | --- | --- |
| Amount of energy required to use the device | \| 1 2 3 \| \| --- \| |
| Length of training (time) needed to become proficient | \| 1 2 3 \| \| --- \| |
| Overall appearance of the device itself | \| 1 2 3 \| \| --- \| |
| Walking speed (getting from A to B quickly) | \| 1 2 3 \| \| --- \| |
| Range (battery life) | \| 1 2 3 \| \| --- \| |
| Ability to climb stairs | \| 1 2 3 \| \| --- \| |
| Ability to toilet while wearing the device | \| 1 2 3 \| \| --- \| |
| Ability to use without arm crutches for balance | \| 1 2 3 \| \| --- \| |
| Ability to carry out daily tasks while standing | \| 1 2 3 \| \| --- \| |
| Portability (e.g. weight, ability for the device to be dismantled for transport) | \| 1 2 3 \| \| --- \| |
| Ability to use getting in and out of a car | \| 1 2 3 \| \| --- \| |
| Ability to walk on sloped or uneven surfaces | \| 1 2 3 \| \| --- \| |
| Purchase cost | \| 1 2 3 \| \| --- \| |
| Repair and maintenance costs | \| 1 2 3 \| \| --- \| |
| Minimizes risk of falling while walking | \| 1 2 3 \| \| --- \| |
| Comfort (e.g. heat, moisture, pain, stability) | \| 1 2 3 \| \| --- \| |

# Exoskeleton questions

# 20. On the following questions, please indicate whether you strongly disagree to strongly agree with the statements:

|  | Strongly Disagree | Disagree | Neutral | Agree | Strongly Agree |
| --- | --- | --- | --- | --- | --- |
| The idea of a powered exoskeleton is a good idea. |  |  |  |  |  |
| If it were made available to me, I would use this technology. |  |  |  |  |  |
| I like the way an exoskeleton looks, based on the video provided |  |  |  |  |  |
| I would feel self-conscious using this technology in public. |  |  |  |  |  |

# 21. What do you feel would be a reasonable price for this technology?

|  | 0-9,999 USD |
| --- | --- |
|  | 10,000-19,999 USD |
|  | 20,000-29,999 USD |
|  | 30,000-49,999 USD |
|  | 50,000-100,000 USD |
|  | Over 100,000 USD |

# End of Survey

Thank you for participating!

# Questions for Health Care Professionals

# 3. What is your age ?

|  | Under 18 |
| --- | --- |
|  | 18-24 |
|  | 25-34 |
|  | 35-44 |
|  | 45-54 |
|  | 55-64 |
|  | 65 or Above |
|  | Prefer Not to Answer |

# 4. What is your gender?

|  | Male |
| --- | --- |
|  | Female |
|  | Transgender |
|  | Other |
|  | Prefer not to disclose |

# 5. What country do you currently live in?

(Drop-down menu of countries)

# 6. What is your current profession?

|  | Equipment Vendor |
| --- | --- |
|  | Occupational Therapist |
|  | Orthotist |
|  | Physician |
|  | Physiotherapist |
|  | Other (please specify) ______________________ |

# 7. How long have you worked in your field?

|  | Under 1 year |
| --- | --- |
|  | 1-4 years |
|  | 5-10 years |
|  | 11+ years |

# 8. Approximately how often do you work with clients with mobility impairments?

|  | Everyday |
| --- | --- |
|  | Once a week |
|  | 2 to 3 times a month |
|  | Once a month |
|  | Less than once a month |

# 9. Please select the highest level of education you have completed.

|  | Primary or elementary school |
| --- | --- |
|  | High school |
|  | College or trade school |
|  | University undergraduate |
|  | Post-graduate |
|  | Doctor of Medicine (or equivalent) |
|  | Other (please specify) ______________________ |

# Exoskeleton questions

# 10. How familiar are you with powered exoskeleton technology?

|  | I have never heard of it (completely unfamiliar) |
| --- | --- |
|  | I am somewhat familiar with the concept |
|  | I am very familiar with the concept |
|  | I would consider myself an expert (completely familiar) |

# 11. Have you ever recommended a powered exoskeleton?

|  | Yes |
| --- | --- |
|  | No |

# 12. Have you recommended any other standing device?

(e.g. a standing frame, standing wheelchair)

|  | Yes |
| --- | --- |
|  | No |

# 13. Is walking with an exoskeleton something that interests your clients with mobility impairments?

|  | Yes |
| --- | --- |
|  | No |
|  | I don't know |

# 14. Is walking a goal for any of your clients with mobility impairments?

|  | None |
| --- | --- |
|  | Few |
|  | Most |
|  | All |
|  | I don't know |

# Exoskeleton Questions

The next set of questions has to do with exoskeleton technology. Please read the description and watch the video below, then answer the following questions regarding your perspectives on the device as shown.

A powered walking exoskeleton is a mobility machine consisting primarily of an outer framework worn by a person, and a powered system of motors that delivers energy for limb movement. The purpose is to improve or replace standing or walking movements for people with mobility impairments. Most current designs require the use of arm crutches for stability, and the user can initiate movement either with hand controls, or using the position of their upper body.

Research has shown health benefits associated with standing and walking for people with a mobility impairment, such as improved: bone density, bowel and bladder function, respiration, and circulation. As well, standing and walking can reduce spasticity and tone, help to prevent pressure sores and contractures, and enhance independence and psychological wellbeing.

*Note: The video is silent.

Exoskeleton Video from Claire Parker on Vimeo: http://vimeo.com/81864531

# 15. Considering the exoskeletons shown above, would you recommend an exoskeleton for...

|  | Yes | No |
| --- | --- | --- |
| Health benefits (e.g. improved circulation, improved bone density, decreased spasticity)? |  |  |
| Rehabilitation purposes (e.g. becoming better at walking)? |  |  |
| Functional day to day tasks? |  |  |
| Social interaction? |  |  |

# 16. Are there any other reasons you would recommend an exoskeleton?

# Exoskeleton questions

Current exoskeleton designs (such as those shown in the video) have a number of limitations. For example, slow movement speed, battery capacity (i.e. travel range), portability, and ease of donning/ doffing pose challenges for its use as a mainstream mobility device. As technology continues to improve, it may be possible to improve on many of these considerations.

For the next set of questions, please consider both current and potential future designs; for example, which factors would be most important to improve upon.

# On the following scale from very unimportant to very important, please rate the importance of each of the following factors in deciding whether you would recommend an exoskeleton from your perspective as a professional.

# 17. Usability and Aesthetic Considerations

|  | Very unimportant | Unimportant | Neither important nor unimportant | Important | Very important |
| --- | --- | --- | --- | --- | --- |
| Ease of putting on/taking off |  |  |  |  |  |
| Amount of energy required to use the device |  |  |  |  |  |
| Length of training (time) needed to become proficient |  |  |  |  |  |
| Overall appearance of the device itself |  |  |  |  |  |

# 18. Functional Considerations

|  | Very unimportant | Unimportant | Neither important nor unimportant | Important | Very important |
| --- | --- | --- | --- | --- | --- |
| Walking speed (getting from A to B quickly) |  |  |  |  |  |
| Range (battery life) |  |  |  |  |  |
| Ability to climb stairs |  |  |  |  |  |
| Ability to toilet while wearing the device |  |  |  |  |  |
| Ability to use without arm crutches for balance |  |  |  |  |  |
| Ability to carry out daily tasks while standing |  |  |  |  |  |
| Portability (e.g. weight, ability for the device to be dismantled for transport) |  |  |  |  |  |
| Ability to use getting in and out of a car |  |  |  |  |  |
| Ability to use on sloped or uneven surfaces |  |  |  |  |  |

# 19. Practical Considerations

|  | Very unimportant | Unimportant | Neither important nor unimportant | Important | Very Important |
| --- | --- | --- | --- | --- | --- |
| Purchase cost |  |  |  |  |  |
| Repair and maintenance costs |  |  |  |  |  |
| Minimizes risk of falling while walking |  |  |  |  |  |
| Comfort (e.g. heat, moisture, pain, stability) |  |  |  |  |  |

# 20. On the previous page, you ranked the following factors as "important" or "very important" when considering whether or not you would recommend an exoskeleton. Please select your top 3 most important factors from this list.

Rank these 3 factors as 1 (the most important) to 3 (the least important) and leave the rest blank.

| Ease of putting on/taking off | \| 1 2 3 \| \| --- \| |
| --- | --- | --- |
| Amount of energy required to use the device | \| 1 2 3 \| \| --- \| |
| Length of training (time) needed to become proficient | \| 1 2 3 \| \| --- \| |
| Overall appearance of the device itself | \| 1 2 3 \| \| --- \| |
| Walking speed (getting from A to B quickly) | \| 1 2 3 \| \| --- \| |
| Range (battery life) | \| 1 2 3 \| \| --- \| |
| Ability to climb stairs | \| 1 2 3 \| \| --- \| |
| Ability to toilet while wearing the device | \| 1 2 3 \| \| --- \| |
| Ability to use without arm crutches for balance | \| 1 2 3 \| \| --- \| |
| Ability to carry out daily tasks while standing | \| 1 2 3 \| \| --- \| |
| Portability (e.g. weight, ability for the device to be dismantled for transport) | \| 1 2 3 \| \| --- \| |
| Ability to use getting in and out of a car | \| 1 2 3 \| \| --- \| |
| Ability to walk on sloped or uneven surfaces | \| 1 2 3 \| \| --- \| |
| Purchase cost | \| 1 2 3 \| \| --- \| |
| Repair and maintenance costs | \| 1 2 3 \| \| --- \| |
| Minimizes risk of falling while walking | \| 1 2 3 \| \| --- \| |
| Comfort (e.g. heat, moisture, pain, stability) | \| 1 2 3 \| \| --- \| |

# Exoskeleton Questions

# 21. On the following questions, please indicate whether you strongly agree to strongly disagree with the statements.

|  | Strongly Disagree | Disagree | Neutral | Agree | Strongly Agree |
| --- | --- | --- | --- | --- | --- |
| The idea of a powered exoskeleton is a good idea. |  |  |  |  |  |
| If it were made available, I would recommend this technology. |  |  |  |  |  |
| I like the way an exoskeleton looks, based on the video provided |  |  |  |  |  |
| I think users would feel self-conscious using this technology in public. |  |  |  |  |  |

# 22. What do you feel would be a reasonable price for this technology?

|  | 0-9,999 USD |
| --- | --- |
|  | 10,000-19,999 USD |
|  | 20,000-29,999 USD |
|  | 30,000-49,000 USD |
|  | 50,000-100,000 USD |
|  | Over 100,000 USD |

# End of Survey

Thank you for participating!
