# Supplementary material for: A survey of stakeholder perspectives on exoskeleton technology
Source: J Neuroeng Rehabil. 2014 Dec 19;11:169. doi: 10.1186/1743-0003-11-169 (PMC4320449; doi:10.1186/1743-0003-11-169)
Supplement: Supplementary file 2 — Additional file 2: Responses to open-ended questions. (DOCX 46 KB) [file 12984_2014_700_MOESM2_ESM.docx]

| Healthcare Professional responses to the question – “Are there any other reasons you would recommend an exoskeleton?” |
| --- |
| Increased self-esteem |
| Emotional and psychological benefits Goal setting |
| Work |
| Psychological benefits of standing and walking. |
| You get to look like Ironman :) |
| Doesn't look functional at all. Will end up in the closet and they will jump into wheelchair and be there in a quarter of the time! For training, ASIA A and B not going to get back to walking from this. For C and D, way more expensive than weight supported walking or pool therapy |
| Quality of life/ goal setting/ adventure/ independence building |
| Psychosocial reasons. Fulfilling a desire to return to walking. |
| It would definitely be appropriate for individuals who are still coping with the loss of mobility (for whatever reason)... Possibly much needed "hope" or simply positive psychological benefits. |
| Improved psycho-social well being |
| For people who would like to walk for short distances. |
| Potentially to provide/assist a patient with confidence if they have a fear of falling. |
| Assist with ticketing and other adls |
| I think it would improve peoples mental state giving them the ability to stand again and feel "normal". |
| We have the technology, lets put it to good use. I am not only a vendor of wheelchairs, including power standers, but have a disabled son who has used a chair since age three. Feel free to contact me as needed. This could be a life changing piece of equipment for a great many people. Keep up the good work |
| Psychological well being |
| It gives user a sense of achievement. |
| Only above as per the clients goals. I woudln't assume social interaction would require standing and walking in most cases, but I would understand if it was required for a particular client. The first two reasons (health benefits and rehab purposes) are the most likely and I would expect the most frequent, as only a client that meets very specific criteria would find one useful for day-to-day funtional activities. |
| Body image, to improve mood |
| Yes , ability to performance task for independent living in some cases. |
| Improved quality of life and decreasing risk of orthostatic hypotension |
| Capacity for employment |
| Motivation during the rehab process. It would be more exciting for a patient to use and exo skelton during therapy to walk somewhere instead of on a treadmill, like the lokomat or ther similiar devices. |
| To improve the ADL's task |
| If I can see how it would give a client more independence, I would recommend it. |
| Sense of overall well being and viewing others at eye level. |
| Endurance |
| Employment |
| Need more info on the device! |
| Healthcare Professional responses to the question – “Are there any other reasons you would recommend an exoskeleton?” |
| It may contribute to an increase in self esteem and a decrease in depression. |
| Improve core strength and trunk control |
| Neuro feedback. Mind body connections. |
| Psychological health of the user |
| Improved self esteem and reduced sense of disability. |
| Personal wellness, confidence and dignity |
| Mostly for health benefits and rehab, it's too bulky still and inaccessible for using day-to-day or socially. I also think it would be more natural to interact with someone in a manual chair than in an exoskeleton. |
| It increases a level of self confidence, gratification (I believe) by increasing their independence and decreasing their dependence on others. |
| As I indicated in the survey. I am a family member. My daughter (age 34) has quadriparesis from her cerebral palsy. She walks with a very unsteady, guarded gait and uses a power chair in community. Basically her right leg is like a crutch. Dr. Tsui at UBC has injected Botox to allow her right foot to flex upward a little more. This has helped with tripping. We have always dreamed that there could be a device that would give more function to her right leg so that she could walk safely for longer distances. This would be good for fitness and health as well as functioning in daily living. She loves to walk. Right now she holds someones arm which is not practical. She walks inside her condo but has a bad fall at least twice a year and many close calls. |
| Accessibility |
| Not sure of my diagnosis |
| Not sure what kind diagnosis. |
| No |
|  |
|  |
|  |
| Wheelchair users’ responses to the question – “Are there any other reasons you would be interested in using an exoskeleton?” |
| Work!! |
| Just to stand up once in a while. |
| It's cool and I want to be Ironman. I want to walk on nature trails. To reach things in house To converse standing up at social events To beat up Aliens. |
| I think it is a great tool to bring awareness to sci research. |
| Amazing to be able to walk and stand after not doing it for 8+ years! My mom would cry! Not really remembering how tall I might be! Injured at 13 y.o. Easier to find an apartment and be social! And all or any of the health benefits! So many reasons! Can it walk through the snow??? Easier to wear a warm coat in the winter while standing too! |
| I am not sure if one can become faster by practicing but for me I would only probably use it to improve the above mentioned. I feel it looks cumbersome and slow not practical for everyday tasks. |
| I would love to use an exoskeleton for daily uses, as well as to exercise the muscles I can no longer use. I have been thinking about something like this that may help me in gaining strength back in my legs. Wow, Please consider me in any further tests, or anything possible. Thank you very much! |
| Wheelchair users’ responses to the question – “Are there any other reasons you would be interested in using an exoskeleton?” |
| Yes, to embrace my husband and children standing up! With their arms fully around me. I would love to go on long walks with my husband and kids again like we used to. Try doing the dishes, cook delicious meals for my family, Enjoy my life again, and walking up and down the stairs in my own beautiful home. See parts of my home that I can't (basement). I would be able to watch my daughter play hockey again by actually sitting in stands and not at ice level. Just to have my freedom back, this would give me a second chance at living my life to the fullest. This would be a dream come true! |
| Regaining of access to objects or activities at a higher (vertically) level. Access to areas a wheelchair wouldnt be able to navigate. |
| To relieve pain when trying to walk. |
| To relieve pressure on my rear end legs and back from sitting all day. To regain eye to eye contact with my peers |
| I would love to have the opportunity to use the exoskeleton because I would bring me greater mobility and ease my current rehabilitation process and transition from a wheelchair to crutches. |
| Exercise...to stretch ... |
| Having the ability to walk independently would be nice! |
| Quality of life |
| I feel that it would improve my quality of life by decreasing my dependence on others to help me get around, as I can not push myself in the wheelchair all day. However, it does not appear to be very friendly in carrying a drink or a shopping bag, and may in fact hinder your ability to multi-task while walking. Retrospectively, a wheelchair has some of the same hindrances, unless you have accessories, such as a cup holder. |
| I am very interested in the exoskeleton, because I have normal feeling in my rear end. So sitting in my chair all day just kills me. Making me to take half a Vicodin at night so that I don't get up all night peeing. Pain makes me have to pee a lot. Plus no more pressure sores. The pain all limits me when it hurts so much that I have to get off my butt. |
| To regain eye level communication. |
| Making progress in appropriate tools for revalidation |
| Would love to meet others standing face to face again. We also have a new nonprofit therapy center Destination Walk, Inc. In NW Arkansas were we see clients all say long who have sci. Would be awesome for them to see the new technology! |
| No |
| It would be awesome to get up and move around whenever you wanted to and try to spend less time in a wheelchair. |
| I can think of a billion other reasons, but why don't we just say an exoskeleton is a billion steps closer to being able to perform more skeletal functions over a wheelchair. |
| Exercise |
| For exercise purposes. |
| More independence in getting around a community not structured for wheelchair users |
| It would allow me to be more functional both at home and in the community. |
| Being upright. Going for a small walk with my husband and/or children. Just the feeling of being upright. Health benefits of walking again. |
| To be a voice advancing wearable technology. Fun to shape early stage technology in a human centric and consumer friendly way. Much more to come! |
| Wheelchair users’ responses to the question – “Are there any other reasons you would be interested in using an exoskeleton?” |
| Curiosity. |
| Get more involved in exercise program |
| Psychological help, the feeling of standing improves emotional security. |
| Change my life and walk again. |
| Psychological |
| My left arm is 100% paralyzed so your model would not work for me. No propreoception or sensation in right arm. |
| In my situation, I would primarily be more interested in using an X0 skeleton for my upper body. People take for granted how important it is to have use of your arms. I can't even consider a system like the one shown above. |
| I would love to try one but I am a quadriplegic with no use of arms so I don't think it would benefit me |
| I would have loved to have used this right after my injury, but it's impossible now. I'm 30 yrs post sci, my bones would shatter like china plate.I wouldn't have -4.5 bone density in my hips and worse in my knees and legs. |
| To fulfill all my necessary tasks, to be among all.to feel the freedom, to acquire comfort, to live easily. To feel normal. To be happy. |
| I would like to use it for mobility issues, health issues with increased circulation and to be able to feel standing upright. |
| Yes, " walking" again learn my limits of thinks to do |
| Independence |
| I am a wheelchair user and i want to rehab in my social life, ex.to play in the theatre |
| Out door activities fishing hunting etc |
| If I could get the same health benefits from simply standing, I wouldn't see the purpose of using an exoskeleton. I can get places faster (and more safely) using my wheelchair. |
| Just to be able to stand again would bring me to a more normal life, like I had 5 years ago. So many place I would like to go and friends I would like to see all have stairs. This would reduce the obstacle that now prevent this. |
| I would be interested to helping to retrain my neuropathways to "relearn" how to walk. Too often emphasis on new technologies and treatments focus on those considered in the "acute" phase on injury. However, research has shown those with long term/chronic injuries can benefit and gain improvement no matter how long ago they were hurt. In addition, I feel that my occupation (government affairs work in DC) will allow me to help expose and educate those in my community to understand the ongoing need for research and funding for those like me, who will benefit from such technology. |
| Chronic debilitation, which is worsening |
| Besides the reasons mentioned above, be Able to play with my daughter as I never could do, because when I suffered the assault, that left me paralyzed, my daughter was with only 8 months and now she is with 6 years old and walk with her and my wife in places that, here in my country, is impossible with wheelchair. Other reason is Look and be seen by other people for the same plan at the same height improves self esteem. Thank you for the opportunity to dream again! |
| Improve my condition, better walking. |
| Bone density |
| Due to arthritis i can not put any pressure on knee and can not straight it out |
| Wheelchair users’ responses to the question – “Are there any other reasons you would be interested in using an exoskeleton?” |
| Very interested of walking again i even. Come the last 2 yearsand work with a trainer at least 3 monts at the celebration center also work with a physio therapist the last 8 years after my accident. Also every day standing up on my machine at home also doing bicycle call motomed very good turning the bike residence1and 2 dont forget im a retiree and avilable any time thank. You |
| For personal self esteem. |
| To possibly advance the technology, and to help myself and others. |
| Any other health benefits make me interested that much more. I miss looking and conversing with a person eye to eye. Would help me mentally and give me confidence. |
| Overall Benefits to my body. |
| Not certain if it would cause more pain or decrease the pain |
| Maybe years ago but now i would be afraid of injury now at 28yrs post and i have a bad knee and the bone mass that has been lost over the years , i used to stand with braces but stop that 14yrs ago. |
| Walk inside my house to see a view/balcony |
| Nope, just want to walk, and if I should use this, well be it, the thing is the financial situation, if you now what i mean... |
| Great exercise! |
| I am a C4 central cord with the ability to ambulate but spastic. I envision as I age if I develop less mobility due to stability it might make it able for me to continue functioning better. |
| No |
| I use leg braces for exercise and health reasons but cannot use them very long. The exoskeleton would allow me to stand longer. |
| Exercise, Athletics, Mobility to access all means of transportation including airplanes and helicopters. |
| Becouse my body will be have so much more benefits and restore more soon of course. |
| Pride and just to feel more normal again. |
| Just being able to do more,reach more etc... I like working on my car and can get to most of it as long as its not at the top or middle! |
| To be able to look people in the eye. Posture |
| I guess this falls under functional day to day tasks, but also job related tasks. Sometimes being seated and having my lap & feet in my way limit my ability to do things. |
| For all purposes of just feeling normal again. The psychological benefit would be enormous too. |
| Psychological reasons (make a wheelchair user feel "normal" again. |
| Since I can't use my fingers/Hands, I'm not sure that I would use it other than for exercise. |
| I am a human factors engineer and innovator. |
| To feeling like I did before my SCI, to be able to walk instead of using my wheelchair, and to feel more confident in myself. |
| I am a writer, and I am interested in writing about the experience of using an exoskeleton, and the process of starting to walk again after decades of using a chair. |
| My rehabilitation has been improving as far as walking and feel this would bring me that much closer to my goal of independently walking |
| Wheelchair users’ responses to the question – “Are there any other reasons you would be interested in using an exoskeleton?” |
| It might assist in improving my mood being at a another person' eye level rather than having to sit and stare up at them all the time. |
| This is exciting! Rehabilitations would be my primary use. |
| Just to be able to walk |
| To be able to "walk down the aisle" shake someone's hand normally, or just be looked up to again as well as look down again! |
| I have extreme pressure sore issues |
| Go places where there are only stairs |
| Outdoor activities such as walking on uneven ground |
| To be able to walk with my grandchildren |
| Hope |
| I've used the eksobionic piece with forearm cruches on "Pro Step" with at a physiotherapist/trainer from the Ekso company in the states, and most often times doctors who were learning how to use this machine would stand behind me to keep me from falling. I've used it 7 times and walked a total of over 2000 steps. The first time Dr. Jamie Borisoff took video of me taking my first steps in 10years on my tablet, plus he took a picture of his shoes. CTV also did a piece on it with me called the bionic man, paraplegic man walks again. It's on the ctv website. Whenever the studies have been given the approval and need people to be given trials, count me in. The feeling of normality is important in day to day living, but functionality is also important. I just want to see the benefits first hand and would relish the opportunity to take part in this research. |
| The sheer independence of life! |
| I've been in a wheelchair for 38+ years now, so I'd use it as much as possible. I know the health benefits of just standing, because I'm in excellent health, so I know the additional benefit of walking would improve my health even more! |
| Being in a wheelchair sucks!!!!!!!!!!!!! |
| Basically I would love to see the ekso-skeleton technology advance to the point where it can completely replace a wheelchair because after more than a decade it is becoming hard on my spine to be forced into the sitting position for the majority of every day. I have used Ekso Bionics (Richmond CA) device extensively at their research facility and in my home as well. Unfortunately, it does not yet have the versatility to allow the user to bend over beyond a very limited range to use a sink, stove, refrigerator, oven, some cabinets, other kitchen appliances etc. Ditto for bathroom fixtures. Can't get into a car seat either at this point. So it's great for walking, but that's about it for now. Would love to see some group engineer in the ability to flex, bend and crouch through a range of motion without falling in order to function fully in all aspects of normal general life. And advance the device to the point of it being possible to use without arm supports (crutches, braces) so arms and hands could be free to be used for activities like cooking while standing etc. |
| I cannot do steps now and I would love to be able to climb a few steps into my son's home. |
| Just feel that my body needs movement before i lose all of my muscle tone. |
| I would be more interested in using one that incorporated functional electrical stimulation to help reverse muscle atrophy. I doubt the exoskeleton alone would do much for that problem. |
| I used to work as an X-ray technologist and I believe with an exoskeleton that I could return to my job. |
| Business meetings it would be nice to be eye level with people again when standing around in meetings. The health benefits alone are worth it and with improved circulation it could save a country billions of dollars in medical bills in regards to pressure soars. Trust me 6 years since my accident and i am SICK of sitting. |

| Wheelchair users’ responses to the question – “Are there any other reasons you would be interested in using an exoskeleton?” |
| --- |
| Emotionally I would feel more hopeful about chronic disease, and taking action. |
| Empowerment |
| I use a mobility assist when I am afraid of falling and injuring myself. While seated in a wheelchair, I am not using my body and I fear using the abilities I currently have. I am not afraid of looking strange or of being slow. I want to use my body so that my body systems work best. I currently use walking poles and a walker when the terrain permits and resort to a wheelchair only when I will need to walk for a long time or when the terrain is uneven and/or dangerous. |
| To go places a wheelchair will not allow me to. |
| No |
| I don't think so because I don't have the arm strength or hands muscles to use the walking canes. |
| How can any para or quad use one of these unless they are newly injured. My doctor would snap if I told him I was going to "stand" even if you claim there is no weight bearing. I do not believe the benefits (asyou pointout above) of walking can be tied to a exoskeleton. I think you are dreaming and dreaming in technicolour. It is absurd to play these kinds of hopes to a technology that has zero proof that it will do anything other that pour research dollars into it. There is no proven evidence of quality of life enhancement as far as I have seen. I have seen the Argo and I will tell you this - it may be emancipating at first but to suggest you can "walk" with it is bizarre. A snail will fly past you. If I need a cup on a high shelf, i'll ask my wife or kid to reach it for me. Oh, and who has 100,000 euro laying around to buy one? Have you considered the pressure sore issues that could result and that an unspecting person can get with one of these things? You guys watch too many Christoper Reeve home movies of him flailing about on a rope suspended from the ceiling (passive walking indeed). Interestingly, didn't he die from compications from a pressure sore? Could he have gotten it from his weird machine? |
| No |
| I would love to use such a device for the health benefits, but it's clearly not designed for anyone with a high cervical injury. |
| Like everything that has a medical need such as wheelchairs, how much does it cost, how durable is it and how long does the battery last? - I can imagine that the cost will be substantial and I'm really curious if you fall, what can and will go wrong? |
| To help further research. |
| Walk my daughter down the isle |
| Would be good for the body overall |
| I have no balance and cannot grasp canes or assistive devices so would need other means of keeping me up right |
| Recreation -/sports-baseball Dancing |
| First I no longer have one leg, knee replace can't be done now yes if I could have the knee operation use the exoskelleton to rehab properly so that I may wear a prothestic again yes please count me in..I am not independent enough using chairs living alone even with a service dog I struggle to be able to do many things cause I can't carry a power chair with me or afford a making vehicle changes of 8,000 and a 5800 dollar smart drive and still limited to getting to my car in winter conditions..I have become a shut in...this device could eleminate such cost if it can operate independently and not with an attatched person that hinders me now for costs of help..I am selling my home to afford independent freedom but the market is too soft and I may be trapped in my home not attaining wage earning needs to buy independent items do to cost. Count me in if one can volunteer. I am not athletic and mobility is what is going to be the cause of my death and cost to the government if I can't make my needed goals and wants. |

| Wheelchair users’ responses to the question – “Are there any other reasons you would be interested in using an exoskeleton?” |
| --- |
| Gait trainer. |
| Used a standing devise at gf strong and always wished for one, for at home use |
| Better bowel,bladder function,gain strength in my legs,hopefully it helps spasticity |
| Mental health benefits (e.g. Improved feeling of equality with able-bodied peers, having face-to-face conversations in a standing position, ability to complete tasks I was unable to from a seated position, etc.) |
| I cannot see being interested, in my current position, I would need to lose weight for 1 thing first. |
| Being able to manage stairs alone or with slight assistance. NOTE: this includes going down stairs. Most videos I have seen of exoskeletons only show people going up stairs, which is much easier and less scary than going down. |
| Mainly for health reasons, bladder and bowel control, increase circulation as I am intolerant to cold, my feet swell from sitting in a wheelchair for long time, weight gain due to decreased mobility |
| No |
| I do walk with fore arm crutches sometimes, my legs get tired easily. I can't stand with my knees straight at all. This would improve my walking a great deal. |
| Yes! To use the washroom, washing and shaving. |
| Improve balance if possible. |
| I don't like using wheelchair all the time. |
| Not sure |
| Cool |
| Curious to know if it would work for people who have difficultyl bearing weight. |
| For the freedom and to just go out walking again to enjoy the outdoors but also to help develop more muscle. |
| It gives me joy to walk even with total assistance for short periods of time. Emotional health. |
| A more comprehensive understanding of the sensation of ambulating. I currently use forearm crutches part of the time but only have functional use of one leg. An exoskeleton would provide an opportunity to gain a somatic appreciation of a more typical gait. |
| My concern is I am hemi -plegic, so have use of only one arm; it seems this device requires two arms to balance. As much as I would love to try,& buy one (depending on the price). I don't see how it's applicable to me in my situation ! |
| I lost my ability to stand a year ago and realize all the benefits of a body being upright are multiple and mandatory for better health. Even being able to stand upright and not walk benefitted my well being every day. |
| Sounds great--however, with the little knowledge I have of this device, it seems far more useful for paraplegics and incomplete SCI. |
| Feeling good, increased leisure opportunities with family and friends |
| It would be one step closer to what I had 5.5 years ago. Freedom from the chair. |
| Better health and exercise. |
| Getting back to my life of fly fishing and camping. Seeing people eye to eye again. Feeling somewhat normal again. |
| 40+ years in a chair now for me so I'm most interested in the health benefits. |

| Wheelchair users’ responses to the question – “Are there any other reasons you would be interested in using an exoskeleton?” |
| --- |
| While the technology is great, I am wondering if this will actually work for all wheelchair bound people. Having MD means that my muscles are weakening by the day. I don't have enough finger strength in one hand to even turn the ignition key in my van or zip up jeans. So to put that much pressure on my arms and hands to hold on to arm crutches would be difficult. I would also worry about the pressure on the knees keeping them locked into place and if balance is thrown off it would be that much easier to fall. (sorry if this is in the wrong place, haven't gotten to the rest of the survey yet to see if there is a more appropriate place.) |
| NO |
| To see the world from a different perspective! |
| While I have a healthy spinal cord my spine has been rebuilt frm T11 to S2 twice. After the first surgery I was mobile to some extent but the spine began to collapse.The surgery was done again ant this time I required forearm crutches and later used the char as required. The spine again did not hold up and a few years ago I had to move to a power chair. It is dangerous for me walk even with the crutches. I do stand for brief periods using the forearm crutches I will be 79 in November and keep myself in reasonable shape with stretchy cords. I was involved with DSA for a.few years. |
| More independence and ability to access areas that are now not accessible to wheelchairs. Lessen the burden of care. |
| It has been 16 plus years, like most people in my situation I would do almost anything to be on my feet again. I have been following the ongoing advances that could help to achieve this and would be very happy to participate in any study to further this goal. I think it would be extremely beneficial for a persons mental state. |
| To be more independent!!! |
| To get rid of hauling a chair around |
| One thing your video assumes, is one has use of both arms! I don't. It would be most useful to me as an aid to standing, which I must do to transrer. To stand now I must pull very hard with my one good hand, in order o achive a standing position puting great stress to my caregiver! |
| Depending on competency, I would use it for everything. |
| To get my leg muscles moving again so the don't continue to atrophy. |
| If I had an opportunity to try using it, I would love the ability to stand straighter. I do abdominal strengthening home physio everyday. What if it could help teach my brain to walk straighter. But I already have the skeletal deformities associated with being middle aged with CP. I am able to walk with aids and have only needed a power wheelchair for the past 6 years. Even though currently I can only walk inside, I am very thankful to have the ability I have. |
| Not sure if I would be able to use an exoskeleton. Very limited movements. ??? |
| To move the technology forward |
| Help me to walk longer distances. Help me to improve my posture while walking |
| Better accessibility |
| Exercise |
| Using an Exoskeleton would make it easier to find a job. |

| Wheelchair users’ responses to the question – “Are there any other reasons you would be interested in using an exoskeleton?” |
| --- |
| I have been watching exoskeletons for a number of years now and on the waiting lists for everyone I can find. I've skyped with the people in Australia but theirs - and all the others so far - are so far out of my financial reach i've been unable to use them. I'd like to stand and kiss my husband, I'd like to meet people eye to eye again, I'd like to breathe the air up there. I absolutely understand the health benefits of standing and moving. I fundraised in my community to buy a "Stand N Glide" machine for our community gym and use it as often as I can. |
| Standing and mobility are health enhancers. |
| The transfers I do today are sliding board I am very functional active tetrapelgic c5 c7 incomplete my tasks such as boarding an air plane standing FOR ADL functionality dressing showering getting into higher cabinets. My needs are help in standing as my tricep traces on both limbs my left side is almost normal whereas my right side is much that of a stroke once standing I can walk I do so in aqua therapy once a week I walk bout 1/4 mile in the warm water pool I have excellent trunk balance and buyonacy. The independence involved would be more than a dream....the more I think of it this could increase my mobility as I have been in constant physical fitness since my injury 25 years post I have the ability to rock to stand but my rather large frame makes the move from clinical to independence a hurdle ive encountered. |
| No. I can get up on crutches for about 12 to 15 minutes. |
| If there was one for people of shorter stature that provided a lot of support for the legs or could adapt to unevenly sized legs I could use it for stuff like walking or hiking or going up and down stairs. |
| I have trouble getting out of a chair or wheelchair - this makes it look easier to stand. I have upper body weaknesses so maybe it would help in standing up. |
| The coolness factor! |
| Hopefully encourage neuroplasticity |
| Mobility Freedom |
| There is also the social aspect of standing and conversing eye to eye. That is very important. I am very vocal in my community about the health benefits of standing and health. I did research on all the benefits plus how they played out financially and then put that info into a presentation and did a fundraiser project to purchase a stand n glide for our rec centre gym, another fundraiser for a raised matt to stretch and work out on and then applied for a grant so all the peers in Quesnel could go to the gym for free to be healthy. |
| Just to be able to stand even for a second |
|  |
